# Online Mental Health Animations for Young People: Qualitative Empirical Thematic Analysis and Knowledge Transfer

**DOI:** 10.2196/21338

**Published:** 2021-02-09

**Authors:** Helen Coughlan, David Quin, Kevin O'Brien, Colm Healy, Jack Deacon, Naoise Kavanagh, Niamh Humphries, Mary C Clarke, Mary Cannon

**Affiliations:** 1 Royal College of Surgeons in Ireland Department of Psychiatry Dublin Ireland; 2 Department of Film and Media Institute of Art, Design and Technology Dun Laoghaire Ireland; 3 SpunOut.ie Dublin Ireland; 4 Jigsaw, The National Centre for Youth Mental Health Dublin Ireland; 5 Royal College of Physicians of Ireland Dublin Ireland; 6 Department of Psychology Royal College of Surgeons in Ireland Dublin Ireland

**Keywords:** mental health, public health, mental health literacy, social media, youth, qualitative, knowledge translation, anxiety, bullying, depression, loneliness, internet

## Abstract

**Background:**

Mental ill-health is one of the most significant health and social issues affecting young people globally. To address the mental health crisis, a number of cross-sectoral research and action priorities have been identified. These include improving mental health literacy, translating research findings into accessible public health outputs, and the use of digital technologies. There are, however, few examples of public health–oriented knowledge transfer activities involving collaborations between researchers, the Arts, and online platforms in the field of youth mental health.

**Objective:**

The primary aim of this project was to translate qualitative research findings into a series of online public mental health animations targeting young people between the ages of 16 and 25 years. A further aim was to track online social media engagement and viewing data for the animations for a period of 12 months.

**Methods:**

Qualitative data were collected from a sample of 17 youth in Ireland, aged 18-21 years, as part of the longitudinal population-based Adolescent Brain Development study. Interviews explored the life histories and the emotional and mental health of participants. The narrative analysis revealed 5 thematic findings relating to young people’s emotional and mental health. Through a collaboration between research, the Arts, and the online sector, the empirical thematic findings were translated into 5 public health animations. The animations were hosted and promoted on 3 social media platforms of the Irish youth health website called SpunOut. Viewing data, collected over a 12-month period, were analyzed to determine the reach of the animations.

**Results:**

Narrative thematic analysis identified anxiety, depression, feeling different, loneliness, and being bullied as common experiences for young people. These thematic findings formed the basis of the animations. During the 12 months following the launch of the animations, they were viewed 15,848 times. A majority of views occurred during the period of the social media ad campaign at a cost of €0.035 (approximately US $0.042) per view. Animations on feeling different and being bullied accounted for the majority of views.

**Conclusions:**

This project demonstrates that online animations provide an accessible means of translating empirical research findings into meaningful public health outputs. They offer a cost-effective way to provide targeted online information about mental health, coping, and help-seeking to young people. Cross-sectoral collaboration is required to leverage the knowledge and expertise required to maximize the quality and potential reach of any knowledge transfer activities. A high level of engagement is possible by targeting non–help-seeking young people on their native social media platforms. Paid promotion is, therefore, an important consideration when budgeting for online knowledge translation and dissemination activities in health research.

## Introduction

If health is the goal, biomedical interventions are not the only means to it. A broadened perspective expands the range of health-promoting practices and enlists the collective efforts of researchers and practitioners who have much to contribute from a variety of disciplines to the health of a nation [1].

Mental health is one of the most significant health and social issues affecting young people globally [2-4]. Mental disorders are the leading cause of disability for people aged 10-24 years [5]. Developing mental ill-health during youth places young people at risk of enduring mental health difficulties [6], which are accompanied by a myriad of social, vocational, and relational consequences [2,3]. Thus, promotion, prevention, and early intervention for young people who may be at risk of developing mental ill-health are global health imperatives [7-12]. The Lancet Commission on Global Mental Health and Sustainable Development [13] has recommended a broad range of approaches across multiple sectors to address the global mental health crisis. Included in these are health promotion and the need to hear from those who have experienced mental health difficulties.

Lack of knowledge about features and signs of mental health difficulties (mental health literacy) and how to access support are both associated with mental health treatment avoidance or delay [14]. However, public health campaigns have been shown to be effective in changing both attitudes and intended behavior, including help-seeking [15-18]. Mental health campaigns that promote mental health literacy, personalize and normalize the experience of mental health difficulties, and have a recovery orientation have been found to both reduce stigma and promote help-seeking behavior [15,18-20]. The effectiveness of public health campaigns can be enhanced by ensuring that messages are well-designed and target and reach intended audiences [16]. In the field of youth mental health, the use of digital technologies and web-based platforms has been identified as an essential way of reaching young people and delivering both mental health information and support [13,21]. At least 91% of Europeans aged 16 to 29 years use the internet on a daily basis [22], and evidence suggests that young people are turning to web-based platforms to access health and mental health information and advice [23-26]. Findings from a recent survey of over 19,000 Irish youth suggested that, after family and friends, the internet is where 20% of adolescents and 33% of young adults go to informally seek information or support on mental health [24]. The anonymity, ease of access, absence of financial or educational barriers, and the nonstigmatizing environment offered by web-based mental health platforms have been identified as positive features of web-based mental health information by young people [27]. Thus, the internet is an ideal space for public health knowledge transfer outputs.

In their study, Wetterlin et al [23] found that 72.3% of respondents aged 17-24 years rated access to videos explaining mental health issues as highly important on web-based platforms. Among the many multimedia formats that can be used, animations have particular potential for public health communication [28]. They have the potential to provide strong symbolic representation of concepts. Additionally, as they are often short in length, they are considered to be an efficient way to communicate complex issues succinctly, to promote learning [29], and to influence intentions to change health-focused behavior [30]. Importantly, they also offer the potential to communicate health information across all levels of literacy [31,32]. This is particularly the case for spoken animations, which have been found to be the most effective way to communicate complex health information to people with low literacy levels [28,31]. In their study, George et al [32] found that people’s responses to video animations were overwhelmingly positive, with most perceiving animation to be more engaging and relatable than other information video formats. Pacing, tone, and character rendering were rated as important factors in individuals’ responses to animations.

Although increasingly a requirement of health research funders [33], there is a dearth of published material documenting knowledge transfer activities in the field of youth mental health research. In this paper, we describe a collaborative knowledge transfer project involving the translation of qualitative research findings on young people’s emotional and mental health into online public health animations.

The project was conceived in response to a Knowledge Exchange and Dissemination Scheme funding call from the Health Research Board in Ireland. The scheme supports dissemination activities aimed at the general public or specific subgroups of the general public and is open to existing Health Research Board grant-holders. HC, the lead author, was conducting qualitative research on the lives and mental health of young people as part of a Health Research Board grant-funded PhD. Emergent findings from her research had provided compelling insights into young people’s lived experiences of emotional and mental health struggles. HC recognized that the reach and impact of her research could be increased significantly if the findings could be meaningfully and creatively translated and shared with other young people. This resulted in a successful application for the Youth Mental Health Animation Creation Project by HC.

The aim of the project was to develop engaging and accessible public mental health animations for young people. The project involved a collaboration between research (Royal College of Surgeons in Ireland), the Arts (the Institute of Art, Design, and Technology), and the online youth health sector (SpunOut). The IADT Animation department was invited to join as a project partner because of its previous experience in translating complex and emotive material through animation for health and mental health organizations in Ireland. To maximize the potential reach of the animations, Ireland’s leading health information website for young people aged 16 to 25 years, SpunOut, also joined as a project partner. SpunOut has over 1.2 million unique users per year with an average of 180,000 individuals accessing content per month [34]. The project was conducted in phases: research data collection and analysis; developing narrative scripts using qualitative data; creating and promoting the animations; and collection and analysis of online engagement and view data.

## Methods

### Study Population

Qualitative data were collected from 17 young people (10 male, 7 female) aged 18-21 years from the Adolescent Brain Development study [35,36], a longitudinal, epidemiological, population-based study that has been examining mental health and brain development among Irish youth since 2007. At the time of the animation project, 3 waves of data collection had been completed: (1) a baseline clinical interview study of 211 young people aged 11-13 years; (2) a follow-up clinical interview study of 86 individuals aged 14-18 years; and (3) a nested qualitative follow up study with a subsample of 17 individuals aged 18-21 years. The aim of the qualitative study was to explore young people’s life narratives with a focus on adverse life experiences, interpersonal relationships, mental health and subjective well-being. Findings from the 17 individuals who took part in the qualitative study at follow up 2 formed the basis of the animations.

### Data Collection

For the qualitative study, data were collected using in-depth qualitative interviews. These were conducted by HC from May 9 to July 25, 2016. Interviews lasted between 45 minutes and 1 hour 50 minutes. A semistructured interview schedule was used to explore participants’ early family life experiences, their experiences of adverse or stressful life events, their mental health, their subjective well-being, their relationships with family and peers, their self-perception, their educational and vocational experiences, and their satisfaction with life. These were explored over each individual’s life course. Written consent, which included consent to audio record study interviews was obtained from all participants. Participants were compensated for their time with a gift voucher. Audio recordings were transcribed by an external transcription agency and were subject to a nondisclosure agreement. All transcripts were subsequently checked for accuracy by HC.

Ethical approval was granted by the Research Ethics Committee of the Royal College of Surgeons in Ireland (RCSI REC 1221 March 2016).

### Data Analysis

Interview data were analyzed using narrative analysis. Narrative analysis refers to a suite of methods that focus on the interpretation of individuals’ lives as told in storied form [37,38]. Narrative analysis recognizes that all knowledge is constructed through multiple subjective interpretations of an individual’s lived experiences and involves a dynamic interplay of subjectivity, perception, meaning, and context involving both the individuals who tell their stories and the researchers who listen and interpret those stories [39]. Specifically, as noted by Kirkman [40], narrative theory offers researchers the ability to “both to retain the complexity of the individual lives they study and to investigate multiple interactions among individuals and cultures.” As a method, it focuses on the stories people tell about their life experiences across time, each of which is understood to have specific meaning to the person telling their story [41].

For this study, thematic narrative analysis [38] was used to identify themes within and across individuals’ life stories. Although some forms of narrative analysis focus on both story content and how people tell their stories, the exclusive focus of thematic narrative analysis is the content of the stories that people tell [38]. However, unlike other thematic methods, such as grounded theory, it focuses on maintaining the integrity of individuals’ stories during the analysis rather than on extracting decontextualized themes across cases [38]. Drawing specifically on the work of McCormack [39], we initially analyzed the construction of interpretive life story summaries for each participant. This process involved repeated listening to and reading of the qualitative interviews, during which notes and memos were documented. Each individual’s life story was then mapped visually (in a mind map format and sequentially, from birth to the time of interview) and a life history summary was written for each individual based on an interpretation of each life story as told by each individual and interpreted by HC. Life story summaries were then examined for key themes within each individual’s life story. The analysis method used to identify themes for each individual was that described by Braun and Clarke [42,43]. It involved the generation of inductive thematic codes for each participant based on both the manifest and latent themes across their life stories. These codes were then examined and combined into broader descriptive themes, which included a number of themes relating to participants’ emotional and mental health.

Once coding was completed for each individual, findings were compared across all participants to identify any shared themes across the sample as a whole. For the animation project, we focused only on thematic findings relating to the mental health of individuals during their midadolescent and early adult years. The rationale for this was that the midadolescent and early adult phase of the lifespan is a peak period of risk for the onset of mental health difficulties [6,44]. We wanted the animations to reflect mental health experiences reported by young people during this potentially vulnerable phase of their lives. It also fit with the 16-to-25-year-old target age range of SpunOut. Five dominant mental health themes were identified across participants’ subjective accounts of issues relating to their emotional and mental health during their mid-adolescent and early adult years. These were Anxiety, Depression, Feeling Different, Loneliness and Being Bullied. These formed the basis for each animation.

### Developing Narrative Scripts Using Qualitative Data

With evidence that videos of no more than 2 minutes duration are optimal to maximize viewer attention and engagement [45], we aimed to create 5 animations of between 60 and 120 seconds each. Furthermore, to ensure that the animations reflected the study findings and captured the authentic voices of young people, this phase involved developing composite narrative scripts for each of the 5 animations using verbatim quotes from multiple participants’ interview data.

All individuals who had attended for interview were recontacted about the animation project. Of the 17 participants who had been interviewed, 7 replied. The project was discussed with each and written consent was sought to use quotes from their interviews to create the scripts. All 7 consented. Interview transcripts for these individuals were examined and any relevant quotes pertaining to the 5 animation themes were extracted. In the small number of instances where relevant quotes were not contained in the interview data from these individuals, quotes from other participants from the study were extracted, edited, and modified for inclusion in the script. Linking phrases were also added by HC to optimize the flow and necessary messaging of each narrative script.

Each script was written as a first-person account following a similar narrative arc based on social cognitive theory [1]. From a social cognitive perspective, positive health behaviors and behavior change are only possible when individuals understand health behaviors, have a belief in their capacity to control their health behaviors, and hold expectations about the possible outcomes for their actions [1]. For example, Meyerowitz and Chaiken [46] found that public health communications that enhanced individuals’ sense of self-efficacy to take action in relation to their own health behaviors were most effective. Each script begins by describing the experience and how it feels, including the emotional, cognitive, physical, social, and relational aspects of the experience. Following this, the script incorporates ambivalence on the part of the young person, capturing young people’s struggles to accept their own suffering and reach out for support. This is in line with existing evidence of ambivalence reported in the literature [47,48]. Each animation then highlights different actions taken to respond to the theme of the script. These include talking to informal supports, engaging in hobbies or other activities, speaking to a trusted adult, and accessing formal counselling and mental health supports. These actions were all reported by participants in the study. They also complement existing evidence on the protective roles of formal and informal supports for young people [49,50], trusted adults in a young person’s life [51], and involvement in meaningful hobbies and social activities [52-54]. Each animation ends with a message that combines hope and realism. Specifically, that the action taken has enhanced the young person’s sense of well-being and connectedness but that attending to emotional and mental health issues is an ongoing process and no single action is a panacea to the existential realities and challenges of the human experience [55]. Thus, in line with evidence on maximizing effectiveness in public health campaigns, the content of each animation incorporated information on mental health literacy and help-seeking, using first-person accounts with a recovery orientation [15,16,18,20]. Scripts (with associated subthemes) can be found in Multimedia Appendix 1.

Once each script had been crafted, all scripts were sent to the research participants who had consented to the use of quotes from their interviews. Scripts were marked for each individual to clarify which quotes had been extracted from their interview data. This was to offer participants an opportunity to withdraw their consent or to remove any quotes if they had any concerns about their anonymity. All participants were satisfied with the scripts as written.

### Animations

#### Creation

Animations were created in collaboration with the Animation program in the Film and Media Department of the IADT in Dublin. IADT is the only institute of art, design, and technology in Ireland that focuses specifically on the creative cultural and technological sectors. This animation project was integrated into the curriculum of third-year animation students in IADT as part of their applied professional practice learning. HC acted as executive producer and executive director for the animations, working with 5 student animation teams who crafted the scripts into the final animations. Students were overseen by DQ, the animation program lead.

**Figure 1:**
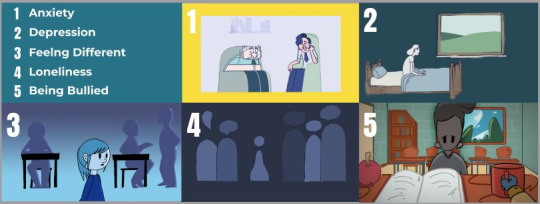
Screenshots from each of the 5 animations.

The collaborative process was designed to maximize the authenticity and potential impact of each animation, while also protecting the research participants’ data. The collaboration combined HC’s expertise in mental health and the animation students’ expertise in conceptualizing, creating and producing animations. It also enabled exploration and discussion of issues such as pacing, tone, and character rendering in each animation [32] (Figure 1). A decision was made by HC to ensure the design style was simple and minimalistic and that character rendering was not overly polished. This was to ensure that the style and rendering of the animations was as congruent as possible with the stories being told. Additionally, in line with evidence on how to maximize animation effectiveness [28,31], first-person narrative voice-overs of the scripts were layered onto the animations. Three of the voice-overs used female actors (depression, loneliness, and feeling different), and 2 used male voice-over actors (anxiety and bullying). The process of cocreating the animations lasted for approximately 4 months.

To promote accessibility, subtitled versions were developed for all animations. Subtitles are essential for individuals who are Deaf/deaf [56] and have also been found to enhance multimedia animation learning in people with attention deficit hyperactivity disorder [57]. Adding subtitles also ensured that the animations could be watched without audio, something of particular relevance to young people’s use of mobile technology. Furthermore, although over 70,000 of people in Ireland speak Irish on a daily basis [58], there is an absence of youth mental health information in the vernacular of young native Irish speakers. To address this deficit, Irish language versions were also developed with support from *Conradh na Gaeilge* [59], a social and cultural organization that promotes the Irish language in Ireland and worldwide.

Once the animations had been completed, they were shown to the research participants who had consented to their verbatim quotes being used. This was to do a final check with those participants that they were satisfied with the animations and to affirm their consent for them to be hosted online. All participants reported being highly satisfied with the completed animations and consented to the launch phase of the project.

#### Promotion

A multimethod approach was adopted in relation to hosting and promoting the animations. First, new content was developed for SpunOut connected to each of the animations. This new content was embedded into the SpunOut website. All existing SpunOut content was also reviewed to identify relevant sections of the website where young people could access further information. Animations were hosted on a unique webpage [60]. Hyperlinks to this hosting page were included in all social media posts to facilitate young people who wished to view more of the animations. All 3 versions of the animations (nonsubtitled, subtitled, and Irish language versions) were also hosted on the SpunOut YouTube channel.

A launch event took place on May 9, 2019, using the hashtag #YMHanimate. Following this, SpunOut engaged in a social media advertising promotion campaign on both Facebook (€300.00, approximately US $362.21) and Twitter (€300.00, approximately US $362.21). The target demographic for the promotion campaign was young people aged 16 to 25 years.

### Collection and Analysis of Online Engagement and View Data

To determine online engagement, analytics data from SpunOut Twitter, Facebook, and YouTube accounts were collected and analyzed for the 12-month period following the launch event. A cut-off view length of 75% or above was used to determine viewing figures across all 3 platforms. One reason for choosing this view length was that analytics data on viewing figures can include views of as little as 2 seconds in length, rendering counts of “any views” unreliable. Additionally, the animation credits accounted for between 9% and 15% of the total view time of each animation. This meant that individuals did not have to watch the full duration of each animation to be exposed to the full content. Data on link clicks (where an individual clicked a related SpunOut content link after watching an animation on social media), costs per view, and viewer demographics could only be extracted from Facebook analytics. Available gender variables were restricted to male, female, and unknown.

## Results

### Views and Link Clicks

Over the period from May 9, 2019 to May 8, 2020, the animations were viewed by 15,848 young people across all social media platforms (based on our criterion of >75% view length). Facebook views accounted for almost two-thirds of all views. Feeling Different was the most viewed animation, followed by Being Bullied and Depression (see Table 1). A majority of views occurred over a period of approximately 2 months following the launch during the social media ad campaign. There were low rates of link clicks on Facebook (ie, when an individual clicked a link to content hosted on the main SpunOut webpage) with just 240 recorded during the period of the social media ad campaign. Further details on impressions and viewing figures are available in Table 1.

**Table 1:** Viewing figures across SpunOut Facebook, Twitter, and YouTube platforms.

Theme	Platform, n^a^			
	Facebook^b^	Twitter^b^	YouTube	Total
Anxiety	1119	594	1133	2846
Depression	2499	104	683	3286
Feeling Different	2447	1005	625	4077
Loneliness	990	81	519	1590
Being Bullied	3382	165	502	4049
Total	10,437	1949	3462	15,848

^a^The number of online users who viewed the animation for a minimum of 75% of its full length.

^b^Figures cover the period from May 9, 2019 to May 8, 2020. Most Facebook and Twitter engagement occurred during the period of the social media campaign.

### Cost Per View and Reach

The cost per view of the animations on Facebook was €0.035 (approximately US $0.042) per young person. This was calculated based on the >75% view length count of 10,437. The cost per reach was €0.003 (approximately US $0.004) per young person, based on a total Facebook reach of 118,142.

### Demographics

Available data from Facebook revealed that 55.1% of those (5750/10,437) who viewed the animations on Facebook were aged 18 to 24 years, 39.9% (4160/10,437) were aged 13 to 17 years, and the remaining 5.0% (527/10,437) were aged 25 years or over. There were higher rates of female viewers than males across all animations and age ranges with the exception of the Being Bullied animation, where higher rates of male views were observed across all age ranges (see Table 2)

**Table 2:** Age and gender of viewers on the SpunOut Facebook platforms from May 9, 2019 to May 8, 2020.

Theme			Age range			
			13-17 years (n=4160), n (%)	18-24 years (n=5750), n (%)	25+ years (n=527), n (%)	All (N=10,437), n (%)
**Anxiety**						
	**Total**		516 (46.1)	506 (45.2)	97 (8.7)	1119 (100)
		Male	160 (31.0)	213 (42.1)	39 (40.2)	412 (36.8)
		Female	353 (68.4)	290 (57.3)	58 (59.8)	701 (62.6)
		Unknown	3 (0.6)	3 (0.6)	0 (0.0)	6 (0.6)
**Depression**						
	**Total**		988 (40.3)	1414 (57.7)	97 (4.0)	2499 (100)
		Male	267 (27.0)	481 (34.0)	38 (39.2)	786 (31.5)
		Female	719 (72.8)	929 (65.7)	59 (60.8)	1707 (68.3)
		Unknown	2 (0.2)	4 (0.3)	0 (0.0)	6 (0.2)
**Feeling different**						
	**Total**		869 (35.5)	1451 (59.3)	127 (5.2)	2447 (100)
		Male	186 (21.4)	341 (23.5)	31 (24.4)	558 (22.8)
		Female	674 (77.6)	1099 (75.7)	96 (75.6)	1869 (76.4)
		Unknown	9 (1.0)	11 (0.8)	0 (0.0)	20 (0.8)
**Loneliness**						
	**Total**		429 (43.3)	534 (53.9)	27 (2.7)	990 (100)
		Male	157 (36.6)	168 (31.5)	13 (48.1)	338 (34.1)
		Female	270 (62.9)	361 (67.6)	14 (51.9)	645 (65.2)
		Unknown	2 (0.5)	5 (0.9)	0 (0.0)	7 (0.7)
**Being bullied**						
	**Total**		1358 (40.2)	1845 (54.6)	179 (5.3)	3382 (100)
		Male	687 (50.6)	1105 (59.9)	100 (55.9)	1892 (55.9)
		Female	664 (48.9)	733 (39.7)	78 (43.6)	1475 (43.7)
		Unknown	7 (0.5)	7 (0.4)	1 (0.6)	15 (0.4)

## Discussion

### General

This is the first knowledge transfer project we are aware of that has translated qualitative research findings on issues affecting young people’s emotional and mental health into a series of bilingual public health online animations. In line with recent recommendations on addressing the global mental health crisis [13,21], the project has given voice to the lived experiences of young people who are struggling with their mental health using a collaborative knowledge transfer process. Our research revealed that experiences of anxiety, depression, loneliness, feeling different, and being bullied were common in the lives of young people during their midadolescent and early adult years. These findings were successfully translated into 5 public health animations through a unique collaboration between the research, Arts, and online sectors. All 5 animations were hosted and promoted by SpunOut [60]. In the 12 months following the launch of the animations, high engagement and viewing numbers were evident across SpunOut social media platforms for all 5 animations, with close to 16,000 views. A majority of engagement occurred during the limited period of the social media ad campaign. The animations exploring Feeling Different and Being Bullied had the highest number of views.

### Comparison With Existing Research and Knowledge

Our qualitative research findings, highlighting young people’s lived experiences of anxiety, depression, loneliness, feeling different, and bullying, are aligned to existing evidence. Epidemiological evidence in Ireland has found that, by the age of 24 years, over 1-in-4 young people in Ireland will have experienced clinical levels of anxiety (26.7%) and depression (28.5%) [61]. More recently, in their study of over 19,000 adolescents and young adults in Ireland, Dooley et al [24] found that 49% of adolescents and 58% of young adults were experiencing anxiety and 40% of adolescents and 58% of young adults were experiencing depression [24]. Anxiety and depression in youth populations have also been recently identified as a significant health issue internationally [62-64].

Our findings on loneliness and feeling different during the adolescent and early adult years complement existing evidence that youth is a key period of risk for loneliness and social disconnection [65-67]. Not only did this emerge as a qualitative theme in the research study, but the Feeling Different animation had the highest number of views. Loneliness and a sense of feeling different are associated with individuals’ needs to explore and find their own identities during adolescence and early adulthood [65]. However, other factors such as culture, environment, personality factors, and gender are also implicated in experiences of loneliness [67]. In the case of gender, females are more likely to report feelings of loneliness and social disconnection, supporting our use of a female voiceover for the loneliness and feeling different animations. The female character representations used in these animations may also have been more congruent with the experiences of females, as reflected in the high prevalence of female views of these animations.

Our finding that many participants in the study had experienced bullying and the high view rate of the Being Bullied animation is consistent with recent Irish data showing rates of 39% and 58% among adolescents and young adults respectively who reported being the victim of bullying [24]. Rates among adolescents are similar to those reported internationally. In their meta-analysis on bullying, Modecki et al [68] found a mean prevalence rate of 35% for traditional bullying and 15% for cyberbullying across the 80 studies in their review. Additionally, our finding that higher numbers of males aged 18 years or older viewed the Being Bullied animation reflects gender trends in the national My World survey [24] of Irish youth where rates of bullying in males increased over time. Specifically, fewer males than females reported being bullied during adolescence (male: 40%, female: 45%) but a higher proportion reported being bullied during their young adult years (male: 61%, female: 57%).

The finding that the animations were viewed almost 16,000 times following their launch demonstrates the potential reach that animations can have within the youth mental health arena. To achieve this, a low budget social media ad campaign was required, and a majority of views occurred in response to this campaign over the campaign period of approximately 2 months. This highlights the value of animations as a medium for knowledge transfer [28,29,31,32] and the importance of budgeting for paid social media promotion to maximize the reach of multimedia knowledge transfer outputs. When proactively seeking mental health information, evidence suggests that young people prefer seeking information from information-based rather than social media websites [23,27,69]. However, for young people who are not proactively seeking mental health information, our high viewing figures during the promotion campaign indicate that social media campaigns may be a particularly effective method to engage non–help-seeking young people with public mental health information. Key to this is following social media trends in order to target those web-based and social media platforms that young people are already using [70].

We anticipated that the animations would enhance mental health literacy in young people, promote disclosure of mental health difficulties, and ensure young people understood how to access informal and formal mental health support. This was based on existing evidence that has shown that public awareness mental health campaigns are effective in achieving both attitude and behavior changes [14,15,18]. For example, an evaluation into the Time-to-Change public mental health awareness campaign in the United Kingdom found that individuals who were simply aware of the campaign reported increased comfort in disclosing mental health difficulties to family and friends and were more likely to seek professional help [18]. Similarly, in their review, Kauer et al [69] found that an increase in mental health literacy was a facilitator of help-seeking among young people accessing information online. A review by Pretorius et al [27] also found that young people used online information and resources to facilitate personal coping responses or as a means to promote informal support seeking behaviors and that the process of help-seeking online could act as a gateway to further help-seeking by connecting young people with information and additional supports. Based on this existing evidence, it is reasonable to hypothesize that, for a proportion of young people, viewing and being exposed to the messaging within each animation will have positively impacted their attitudes toward mental health difficulties, their mental health literacy (for those with low levels of mental health literacy), their willingness to share any current or future mental health concerns, and their willingness to reach out for information and support if they need to.

A final and important finding from this project was the low cost–high yield relationship between what was spent on social media promotion and the level of user engagement and views of the animations. In their systematic review of the use of social networking sites for public health practice and research, Capurro and colleagues [70] highlight that social media and social networking sites offer researchers the fast, easy, and low-cost access to a range of populations, making them an ideal platform for conducting research. Our cost-per-view findings, at a cost of just €0.003 (approximately US $0.004) to reach a young person with one of the videos and €0.035 (approximately US $0.042) to have a young person in our target demographic view the video to completion highlights the potential that social media promotion can offer in supporting impactful knowledge transfer activities targeting known populations at a very low cost. Moreover, our use of this method addresses a key factor that has been identified in maximizing the potential for public health messaging to change behavior: ensuring that messages are delivered to their intended audience with sufficient reach [16]. Additionally, it facilitated access to demographic and engagement data, an oftentimes underused data resource in the field of health-related research [70].

### Limitations

A key limitation in this knowledge transfer project is that, while we were able to identify our target demographic for our Facebook and Twitter promotion campaigns, such promotion activity also relies on algorithms and models that are controlled by each social media or networking site. Thus, our demographic findings relating to the age and gender of user engagement may reflect aspects of the advertising algorithms used rather than solely reflecting gender trends related to the animations. Additionally, the idea to develop the animations was a response to the findings emerging from the research. This meant that the focus of the animation project was on ensuring the animations were embedded and accessible to young people as part of an existing and reputable youth mental health website rather than on collecting data on young people’s responses to the animations or their impact on attitudes, health, or help-seeking. This limited our analysis to the reach of the animations. We were therefore unable to examine the impact of the animations on those 15,000 or more young people who watched them or to interpret the low link click rate in our analytics data. However, in relation to the latter, it is important to note that link-click data were only collected during the short period of the social media and promotion period. The limitations of the reach data in this study highlight the importance of integrating research to evaluate impact and effectiveness when designing public health campaigns such as this. Future research is needed to examine the impact of both outputs, such as our animations, and the effectiveness of targeting young people on social media platforms.

### Conclusions

In line with recommendations for tackling the global mental health crisis [13,21], this knowledge transfer project provides an example of how the mental health research community can engage in meaningful knowledge transfer activities targeting young people on their native social media platforms. By adopting this type of knowledge transfer activity, researchers have the potential to use and translate their findings to make a tangible difference to both individual lives and to overall societal health, beyond what is possible within the confines of traditional dissemination arenas and institutions.

## Appendix

Multimedia Appendix 1 Animation themes, scripts, subthemes, health-promoting issues, and actions.

## Abbreviations

IADT: Institute of Art, Design and Technology
