# Supplementary material for: Online Mental Health Animations for Young People: Qualitative Empirical Thematic Analysis and Knowledge Transfer
Source: J Med Internet Res. 2021 Feb 9;23(2):e21338. doi: 10.2196/21338 (PMC7902194; doi:10.2196/21338)
Supplement: Multimedia Appendix 1 [file jmir_v23i2e21338_app1.pdf]

Multimedia Appendix 1 Animation themes, scripts, sub-themes, health-promoting issues and actions*

THEME	Script	Sub-themes	Health promoting issues and actions	Length [seconds]
ANXIETY	When I was young, everyone called me the worrier kid but I didn't really notice it until after I started secondary school. I was in my head all the time. I just kept over-thinking things and I kept thinking like, if a friend didn't contact me, that friend didn't like me. I'd start making up my own theory about it and it would just go out of control. Even when I was trying to do other things, my head was full of negative thoughts going round and around. It got so bad that I couldn't work out what was realistic thinking and what was irrational. I remember feeling like I couldn't escape it. I got very self-conscious and one day I ended up having a panic attack in school. I felt like my heart was in my throat and it all peaked for about 10 minutes. It was awful. My friends said I should see the school counsellor but I was, like, "There's actually nothing wrong with me I'm just being an idiot, like, it's just all in my head". But, I just couldn't manage the anxiety on my own and, after a few really bad weeks, I made an appointment to see the counsellor. And, even though I didn't want to go, it really helped. Since then, my anxiety still flairs up every now and again but I manage it much better than before. These days, I try not to over-analyse everything and, when I do start to feel anxious, I've learned ways to relax myself and stop my thoughts getting out of control.	Cognitive, emotional and physical aspects of anxiety<br><br>Periods of transition can be times of vulnerability for young people's mental health<br><br>Resistance and ambivalence about help-seeking among young people<br><br>Benefits of help-seeking when struggling with anxiety<br><br>Symptoms of anxiety often continue but can be managed with the right support	Role of peers in promoting help-seeking<br><br>Accessing school-based counselling support	110
DEPRESSION	I used to feel kind of emotionless. At the start, I just felt sad, but after a while I stopped feeling anything at all. It was like I was empty and my whole insides felt like they were sinking. I felt totally numb and blank all the time. I wasn't able to enjoy anything and I stopped going out or seeing my friends. Sometimes, I'd sleep for 16 hours a day. When I was down like that, I felt like I was stuck in my own head– like, everything I thought about in the past and in the future was bad. Even though I tried to hide how bad I felt, my mum could see it and got me to see my local doctor. He thought I was probably depressed and referred me to my local mental health service. When I went to them I just talked about what was going on and they definitely helped me make sense of my thoughts and how I was feeling. After a couple of months I was like, 'OK, like I'm actually enjoying things again' and I suddenly realised I had started to feel my emotions again. It hasn't always been easy since then but, one of the things I've realised is that it is possible to get through depression and to feel ok again.	Cognitive, emotional, behavioural, social and physical aspects of depression<br><br>Emotional numbness as a feature of depression<br><br>Isolation that can come with experiencing depression<br><br>Resistance to sharing how one is feeling<br><br>Getting through depression takes time but is possible with the right type and level of support	Role of parents<br><br>Help-seeking via a General Practitioner (GP)<br><br>Role of GP in gaining access to formal mental health services<br><br>Role of formal mental health services for significant mental health issues	80

FEELING DIFFERENT	Well, I just kind of always thought that everything I did was wrong. I always felt kind of different as well. I guess, everyone has that to some extent. I remember thinking 'Why is it me that has to be different?' And I'd judge myself about it. There were times where I would go like, 'I wish I could just be more like you' and I tried to fit in but I couldn't. And then, you know, I stopped trying so hard to be someone I wasn't. I started doing things I enjoyed doing and got involved in a local music club. It's so different for me there. We always end up sitting around having proper conversation. Everyone is really engaged. It's great. It feels like how it should be. For me, it just feels natural. I used to be exhausted putting on a front all the time but now it's just like I'm myself. I know who I am.	Negative cognitions about self often experienced by young people<br><br>Sense of being different to others<br><br>Feeling under pressure to change oneself to be like others<br><br>Value and impact of accepting oneself	Positive impact of engaging in hobbies and activities that you enjoy<br><br>Positive impact of connecting with like-minded others who accept you for who you are	65
LONELINESS	I used to feel kind lonely, kind of isolated, like the conversation was going on all around me but I wasn't part of it. Everyone had their lives so together. It was like I didn't fit in and I had no idea where I was going. But, no-one ever saw it. So, for a long time, I just pretended I was happy. Then, there was this day when I just burst into tears in my room. I'd probably just been bottling everything up for so long that it all came out there and then. I think not telling anyone just made me more overwhelmed than ever. That's when I realised I needed to tell someone that I was struggling. The first person I told said that they felt exactly the same and, for the first time in years, I didn't feel so alone. Going to the gym or having that one person to talk to recharges me. And, I've a few friends I can ring and say, "look, I'm just not feeling myself today". When I think about all I've been through, it's made me realise that I have people I can turn to.	The subjective feeling of loneliness<br><br>Pretence of happiness often expressed by young people<br><br>Impact of bottling up emotions<br><br>Value of sharing how you feel with others<br><br>Realising you are not alone in how you feel	Benefits of physical activity and hobbies<br><br>Help-seeking via friends<br><br>Positive impact of meaningful connections with others	72
BEING BULLIED	For years I had no self-worth. I was kind of getting bullied in school and that seemed to have a big effect on me. I was always able to make new friends but then they'd kind of dump me or whatever. It was soul crushing. I genuinely felt like everyone was against me. That I sort of didn't have any friends to fall back on. Before I was bullied I don't think I was ever anxious about anything but after it started I remember feeling really anxious going into school. Then, I built up kind of a resentment against the people that I had once considered to be my friends. Finally, I told one of my teachers and I remember the sense of relief I felt that someone finally knew what was happening to me. Just having someone to talk to made such a difference. I ended up doing some volunteer work that summer and when I started and when I was making new friends there, I quickly realised this is what actual friendship is - not getting bullied or slagged or whatever and I was like, "Well I don't really care what those people think anymore". Finding people and friends that I actually connect with has helped me a lot and it's great that I've met people and I'm like, 'Yeah, you get it' kind of thing.	Negative impact of bullying on a young person's sense of self-worth<br><br>Cognitive, emotional, behavioural, social and physical aspects of being bullied<br><br>Positive impact of reaching out to a trusted adult to share what's going on	Help-seeking via a teacher<br><br>Positive impact of engaging in hobbies or volunteer work with like-minded others<br><br>Positive impact of making meaningful relational connections with others	76

***** All animations available to view at www.spunout.ie/RCSI and www.youtube.com/playlist?list=PLxkgc2VmXsQuPdFIS5UjAZ39LzhCbXKBP
